# Understanding human functioning using graphical models

**DOI:** 10.1186/1471-2288-10-14

**Published:** 2010-02-11

**Authors:** Markus Kalisch, Bernd AG Fellinghauer, Eva Grill, Marloes H Maathuis, Ulrich Mansmann, Peter Bühlmann, Gerold Stucki

**Affiliations:** 1 Swiss Paraplegic Research (SPF), Nottwil, Switzerland; 2 Seminar für Statistik, ETH Zürich, Zürich, Switzerland; 3 Institute for Health and Rehabilitation Sciences, Ludwig-Maximilians-University, Munich, Germany; 4 Department of Medical Informatics, Biometry and Epidemiology, Ludwig-Maximilians-University, Munich, Germany; 5 Department of Health Sciences and Health Policy, University of Lucerne, Lucerne, Switzerland; 6 ICF Research Branch, WHO FIC CC Germany (DIMDI) at SPF, Nottwil, Switzerland

## Abstract

**Background:**

Functioning and disability are universal human experiences. However, our current understanding of functioning from a comprehensive perspective is limited. The development of the International Classification of Functioning, Disability and Health (ICF) on the one hand and recent developments in graphical modeling on the other hand might be combined and open the door to a more comprehensive understanding of human functioning. The objective of our paper therefore is to explore how graphical models can be used in the study of ICF data for a range of applications.

**Methods:**

We show the applicability of graphical models on ICF data for different tasks: Visualization of the dependence structure of the data set, dimension reduction and comparison of subpopulations. Moreover, we further developed and applied recent findings in causal inference using graphical models to estimate bounds on intervention effects in an observational study with many variables and without knowing the underlying causal structure.

**Results:**

In each field, graphical models could be applied giving results of high face-validity. In particular, graphical models could be used for visualization of functioning in patients with spinal cord injury. The resulting graph consisted of several connected components which can be used for dimension reduction. Moreover, we found that the differences in the dependence structures between subpopulations were relevant and could be systematically analyzed using graphical models. Finally, when estimating bounds on causal effects of ICF categories on general health perceptions among patients with chronic health conditions, we found that the five ICF categories that showed the strongest effect were plausible.

**Conclusions:**

Graphical Models are a flexible tool and lend themselves for a wide range of applications. In particular, studies involving ICF data seem to be suited for analysis using graphical models.

## Background

In recent years, research in health conditions with high impact such as cancer, cardiovascular, and neurodegenerative diseases has been redefined from simplistic cause models towards a systems approach. This permits complex interactions of multiple components embedded in the cellular machinery of the body, and hence a new understanding of disease and aetiology. Similar to this new approach to genomic and cellular disease mechanisms, macroscopic views on health and disease are changing.

Health is increasingly understood as a complex interaction of functioning with a multifaceted environment. Functioning and disability are universal human experiences in which body, behavior and society are inextricably intertwined [1,2]. In our lifespan we all will experience limitations in functioning due to acute or chronic health conditions, or aging. Modern societies aim to optimize functioning and quality of life through rehabilitative efforts on the clinical, service and policy level [3,4].

Our current understanding of functioning from a comprehensive perspective is limited. The World Health Organization (WHO) has recently made a compelling case to develop human functioning and rehabilitation research in its resolution R114 on 'disability, including prevention, management and rehabilitation' [4]. All substantive articles of the UN Convention on the Rights of Persons with Disabilities refer to specific domains of human functioning. Human rights require appropriate levels of functioning. Significantly implementing a right means to know and analyze all the relevant levels of human functioning in concert with a facilitating environment. The right to education, for example, can only be implemented by enabling mobility, communication, access to appropriate facilities and support. We require, in short, a comprehensive understanding of human functioning. Likewise, the "Rehabilitation Medicine Summit: Building Research Capacity" which was organized by the Foundation for Physical Medicine and Rehabilitation, the American Academy of Physical Medicine and Rehabilitation, the American Congress of Rehabilitation Medicine, and the Association of Academic Physiatrists stated the need to increase investment in human functioning and rehabilitation research [5].

Still, there has been considerable progress into the understanding of functioning. Among the recent developments is the International Classification of Functioning, Disability and Health (ICF; see [3]), and as a consequence the possibility for innovative modeling strategies in that context. The ICF which was endorsed by the World Health Assembly in May 2001 [4] provides a common language for functioning and health. With the ICF it is possible to systematically define the prototypical spectrum of functioning and health domains for specific health conditions, therapy targets, and age groups. Likewise, the association of elements of functioning on the level of single categories can be analyzed beyond the study of the incidence and prevalence of disease.

It is a difficult task to catch the complex associations encountered in human functioning research. This methodological problem has been solved for the domain of genomic medicine by using graphical modeling. Graphical models were identified as a promising new approach to modeling clinical data [6], and thus the systems approach to health and disease. Beyond association, this method has also been developed for estimating causal effects [7].

Based on current work [8], graphical models have potential for a range of applications. Firstly, the dependence structure of complex data can be visualized by graphs thus facilitating intuitive understanding. Secondly, graphical models can be used for dimension reduction of complex data. Thirdly, differences in dependence structures between subpopulations can be clarified. Fourthly, under stricter assumptions, bounds on the (causal) effects of interventions can be estimated from observational data using graphical models. Yet, so far there is no systematic road-map to describe the potential applications of graphical models for the study of functioning.

The objective of our paper therefore is to explore how graphical models can be used in the study of ICF data in order to develop a more detailed understanding of human functioning.

Specifically, our first aim was to develop and improve statistical approaches to the visualization of complex associations. Our second aim was to examine how graphical models can be used for dimension reduction. Our third aim was to examine how differences in association structures between subpopulations can be clarified. Finally, our fourth aim was to investigate the possibility of estimating intervention effects from observational data without knowing the underlying causal structure.

## Methods

### Study design and population

This study was a post hoc analysis of two data sets collected in the context of the ICF Core Set project. Methods for data collection and descriptive analyses have been published elsewhere (see [9] and [10]).

#### Spinal Cord Injury (SCI) data

The multi center, cross-sectional study of patients with SCI was conducted in 14 countries. Individuals were included if they had sustained a SCI with an acute onset, or if they were receiving rehabilitation in the early post-acute situation, or if they were in a long-term context. Individuals had to be at least 18 years old and had to be able to understand purpose and reason of the study. Written informed consent was obtained from all included patients. Individuals with significant traumatic brain injury or diagnosed mental disorders prior to SCI were excluded. Acute onset was defined as injury or disease with the development of SCI within 14 days. The early post-acute context was defined as starting with active rehabilitation and ending with the completion of the first comprehensive rehabilitation after the acute SCI. The long-term context follows the early post-acute context. This working definition was based on a worldwide consensus of researchers involved in the data collection and was approved by the steering committee of the project. In total, 1026 patients were included, of which 22% were female. Patients had a mean age of 42 years. Mean time since injury was 6 years with 46% of the patients being tetraplegic and 54% being paraplegic. Impairment according to ASIA score was complete in 52% of the patients. For subgroup analyses, we used data from 598 patients: 304 from four European countries (Denmark, Germany, Israel, and Switzerland) and 294 from four Asian countries (India, Malaysia, Thailand, and Vietnam). An extensive description of this data set can be found in [9]. We obtained permission to reanalyze the data.

#### Chronic Health Conditions (CHC) data

We used data from a multi center, cross-sectional study involving 1039 patients with chronic health conditions. Individuals were included if they were undergoing inpatient or outpatient rehabilitation in 19 German hospitals and rehabilitation centers and had at least one of the following chronic health conditions: low back pain, osteoporosis, rheumatoid arthritis, osteoarthritis, chronic ischemic heart disease, chronic obstructive pulmonary disease, diabetes mellitus, breast cancer, obesity, chronic widespread pain, depression, stroke. The percentage of female patients was 59%. Patients had a mean age of 53 years. Main diagnoses were low back pain (19%) and chronic widespread pain (12%) followed by breast cancer (12%) and stroke (11%). An extensive description of this data set can be found in [10]. We obtained permission to reanalyze the data.

### Measures

#### ICF

The International Classification of Functioning, Disability and Health (ICF) is a multipurpose classification which belongs to the World Health Organization (WHO) family of international classifications. The ICF contains so-called ICF categories organized in two parts, each consisting of separate components. The first part covers functioning and disability with the components "Body Functions" (coded with *b*), "Body Structures" (*s*) and "Activities and Participation" (*d*). The second part covers contextual factors with the components "Environmental Factors" (*e*) and "Personal Factors". The ICF categories of each component, with exception of the "Personal Factors", which are not classified yet, are hierarchically detailed up to four levels. The hierarchical code system consists of the abbreviation of the component and the chapter number (e.g. *b2 Sensory functions and pain*), followed by the second level (e.g. *b210 Seeing functions*), the third level (e.g. *b2100 Visual acuity functions*) and the fourth level (e.g. *b21000 Binocular acuity of distant vision*). The SCI data comprised 268 second level categories. The CHC data comprised 128 second level categories. The ICF suggests qualifiers which range from 0 to 4 for each category in *b*, *d *and *s *and from -4 to 4 in *e*. ICF codes and descriptions of the ICF categories used in this paper are summarized in Table 1.

**Table 1:** ICF code and descriptions. ICF codes and descriptions of the ICF categories used in this paper.

Code	Description	Code	Description
b152	Emotional functions	d830	Higher education
b280	Sensation of pain	d840	Apprenticeship (work preparation)
b515	Digestive functions	d845	Acquiring, keeping and terminating a job
b860	Functions of nails	d850	Remunerative employment
d170	Writing	d855	Non-remunerative employment
d230	Carrying out daily routine	d860	Basic economic transactions
d330	Speaking	d870	Economic self-sufficiency
d345	Writing messages	d910	Community life
d350	Conversation	d920	Recreation and leisure
d355	Discussion	d930	Religion and spirituality
d360	Using communication devices and techniques	d940	Human rights
d430	Lifting and carrying objects	d950	Political life and citizenship
d450	Walking	s140	Structure of sympathetic nervous system
d465	Moving around using equipment	s150	Structure of parasympathetic nervous system
d470	Using transportation	s220	Structure of eyeball
d475	Driving	s320	Structure of mouth
d570	Looking after one's health	s520	Structure of oesophagus
d610	Acquiring a place to live	s610	Structure of urinary system
d620	Acquisition of goods and services	s620	Structure of pelvic floor
d630	Preparing meals	s630	Structure of reproductive system
d640	Doing housework	s710	Structure of head and neck region
d650	Caring for household objects	s720	Structure of shoulder region
d660	Assisting others	s730	Structure of upper extremity
d710	Basic interpersonal interactions	s740	Structure of pelvic region
d720	Complex interpersonal interactions	s750	Structure of lower extremity
d730	Relating with strangers	s760	Structure of trunk
d740	Formal relationships	s770	Add. musculoskeletal struct. rel. to movement
d750	Informal social relationships	s810	Structure of areas of skin
d760	Family relationships	s820	Structure of skin glands
d770	Intimate relationships	s830	Structure of nails
d820	School education	s840	Structure of hair
d825	Vocational training		

#### Outcome variable

The SF-36 is one of the most frequently used instruments for assessing generic health related quality of life [11]. It contains a total of 36 items organised in eight different categories of scores: Physical Functioning, Role Physical, Bodily Pain, General Health, Vitality, Social Functioning, Role Emotional, and Mental Health. The scores range from 0 to 100 with higher scores indicating better health status. We used the General Health Perception score (ghp) as an outcome for predictive modeling.

### Preprocessing

We used the free statistical software R for all our computations. Both R and all mentioned packages are freely available (see [12]).

#### Handling of missing data

For the SCI data set, we excluded categories which had more than 20% values missing. This resulted in a SCI data set with 200 ICF categories (79 categories from the component Body Functions, 81 from Activities and Participation, 40 from Body Structures and 0 from Environmental Factors). Since in the CHC data set missing values were much more abundant, we only excluded a category if it had more than 50% of missing values. This resulted in a CHC data set with 126 ICF categories (32 categories from the component Body Functions, 47 from Activities and Participation, 15 from Body Structures and 32 from Environmental Factors).

In both data sets, the problem of remaining missing values was addressed by using multiple imputation [13] assuming noninformative missingness. Multiple imputation generates m versions of the original data set, with varying missing value replacements in each version and using information from all other variables to generate the replacement. Simulation studies demonstrated that even with few generated data sets multiple imputation yielded valid results [14]. The SCI data was imputed ten times using multiple imputation (using the option "logistic regression"), while the CHC data was imputed five times (using the option "predictive mean matching"). Different options were used, since the SCI data (after removing category *e*) consisted only of binary variables (see next section), whereas the CHC data consisted of categorical variables with many levels. In the next step, each of these imputed data sets was analyzed using common complete case methods as described below. For computations, we used the R-package "mice" which implements multiple imputation (see [13]).

#### Dichotomization

Because the properties of the qualifiers are not yet evaluated sufficiently, we dichotomized the ICF categories. Each category of the components Body Functions, Body Structures and Activities and Participation was graded with the qualifiers 0 for "no impairment/limitation" and 1 for "any impairment/limitation" The categories of the component Environmental Factors were graded with 0 for "barrier/neutral" and 1 for "facilitator". In the original study generating the SCI data, only the distinction "no impairment" vs. "impairment" (in *b, d, s*) and "barrier", "neutral" and "facilitator" (in *e*) was made. Since category *e *was excluded as stated in the previous section, we didn't have to dichotomize the SCI data set any further.

#### Bootstrap aggregation

Graphical models are susceptible to small changes in the data set leading to large variations and hence unstable results. A common method to enhance unstable procedures is to use bootstrap aggregation [15], which enhances the overall performance of the model building process. Bootstrap aggregation produces several models based on bootstrap replicates of the original data set. The multiple versions are then aggregated. Bootstrap aggregation can stabilize the outcome of a model and enhance accuracy [15]. We generated 10 bootstrap replications. These 10 replications of the 10 imputed SCI data sets and of the 5 imputed CHC data sets generated 100 re-sampled SCI and 50 re-sampled CHC data sets. Bootstrap aggregation was carried out using R-programs developed by ourselves.

Depending on the specific analysis, we aggregated the results on the 50 or 100 data sets as explained in detail below and thus obtained more stable and reliable results.

### Principles of graphical models

Graphical models can be thought of as maps of dependence structures of a given probability distribution or a sample thereof (see for example [16]). In order to illustrate the analogy, let us consider a road map. In order to be able to use a road map, one needs two given factors. Firstly, one needs the physical map with symbols such as dots and lines. Secondly, one needs a rule for interpreting the symbols. For instance, a railroad map and a map for electric circuits might look very much alike, but their interpretation differs a lot. In the same sense, a graphical model is a map. Firstly, a graphical model consists of a graph with dots, lines and potentially arrowheads. Secondly, a graphical model always comes with a rule for interpreting this graph. In general, nodes in the graph represent (random) variables and edges represent some kind of dependence.

An example of a graphical model is the Directed Acyclic Graph (DAG) model. The physical map here is a graph consisting of nodes and arrows (only one arrowhead per line) connecting the nodes. As a further restriction, the arrows must be directed in a way, so that it is not possible to trace a circle when following the arrowheads. The interpretation rule is d-separation, which is closely related to conditional independence. This rule is a bit more intricate and we refer the reader to [16] for more details.

Another example of a graphical model is the so called "skeleton" (of a Directed Acyclic Graph, see [16]) model. The physical map in this model is a graph consisting of dots and lines (without arrowheads). Using this model, we will use the following rules for interpreting a graph: Two nodes are connected by an edge, if and only if the corresponding random variables are dependent if conditioning on any subset of the remaining random variables. Thus, an edge indicates a strong kind of dependence and it turns out that this is useful for estimating bounds on causal effects (also called intervention effects). See [17] for a detailed discussion of this subject.

DAG models are particularly useful for estimating intervention effects. Imagine that a causal system is represented by a DAG: Nodes represent observable variables and arrows represent direct causes. Now assume that we gather data from the causal system by observing it many times in different states and recording the values of all involved variables. The observed data will entail some dependence information among the variables. Since every DAG on the same variables also entails dependence information via d-separation, we could find the DAG that fits the dependence information in the data best. It is a basic fact of DAG models, that we usually won't be able to identify a unique DAG that fits best. Rather, we will find several DAG models that fit all equally well. These DAG models are called "equivalent". The DAGs of the equivalent DAG models have a noteworthy property: When ignoring the arrowheads, they look the same. But some arrowheads point into different directions, i.e., the direction of some edges is ambiguous. It was shown in [17], that under certain assumptions the unambiguous arrows in the estimated DAG models coincide with the true arrows in the underlying causal system. Thus, by estimating a DAG model and under some assumptions, we can get information about the underlying causal structure. This information is contained in the unambiguous arrows of the DAG model. However, the ambiguous arrows don't contain direct information on the underlying causal structure. Hence, estimating a DAG model from observational data gives insight into some aspects of the underlying causal structure, but other aspects will remain obscure. For this reason, it is in general only possible to estimate bounds (and not precise values) on causal effects from observational data.

For estimating (skeletons of) DAG models from observational data, we used the PC algorithm, which is explained in detail in [18,19]. In short, the PC-Algorithm makes a cleverly arranged series of conditional independence tests to transform the data matrix into the skeleton. Subsequently, the algorithm identifies the unambiguous arrows to find the equivalence class of DAG models that fit the data equally well. As independence test we used the Fisher Exact Test for marginal independencies and a simulation based test (implemented in coindep_test in the R-package vcd) for conditional independencies.

Several recent papers have tried to improve the performance of the PC algorithm (see for example [20,21] and [22]). Each of these algorithms can be used as an alternative to the PC algorithm in the analysis we propose.

### Application of graphical models to ICF data

For visualization (i.e. estimation of the skeleton), dimension reduction and comparison of subpopulations we used the SCI data set. For the estimation of bounds on causal effects we used the CHC data set, since a clear response variable and a detailed regression analysis (see [10]) for comparison was available at the time of analysis.

#### Visualization

In order to show the dependence structure of the data we used the PC-Algorithm for finding the skeleton model. The skeleton graph was estimated from the SCI data set. The resulting 100 graphs were aggregated by generating a summary graph, where the summary graph included an edge if this edge was found in the skeleton of at least 20 re-sampled data sets (the number 20 being a conservative choice that reduces random fluctuations but still keeps meaningful, but weak signals). We defined the reliability of an edge as being proportional to the number of re-sampled data sets that indicate the presence of this edge. The thickness of the edges is proportional to their reliability.

#### Dimension reduction

The skeleton can also be used for dimension reduction. Sometimes, a skeleton can be split up into several groups of variables, that are connected within the group, but not connected between the groups. We call such a group a "connected component". It can be shown, that variables of one connected component are statistically independent of variables of another connected component. Thus, any further analysis technique can be applied separately to each connected component. As before, we only report on the summary graph for the SCI data.

#### Comparison of subpopulations

In order to compare the dependence structures of subpopulations, we used the SCI data and estimated one skeleton for the Asian countries and one skeleton for the European countries. Thus, the comparison of subpopulations reduced to the comparison of two graphs. The dissimilarity of two graphs can be quantified using the Structural Hamming Distance (SHD). The SHD between two skeletons is the number of edge insertions or deletions that are needed to transform one graph into the other. The graphs of two regions were judged to be significantly different, when the variation in SHD between regions was larger than the variation in SHD introduced by bootstrapping and multiple imputation. In order to identify the characterizing differences between two graphs, we searched for highly reliable edges which are present in the graph of one subpopulation but are absent in the graph of the other subpopulation.

#### Estimating causal (intervention) effects

Although it is a general postulate that causal effects can exclusively be estimated from experimental studies where an intervention is applied at random, in principle and under certain assumptions (no unmeasured confounders, no measured selection variables and some other, more technical assumptions) it is also possible to estimate bounds on intervention effects for Gaussian variables from observational data, even if the underlying causal structure is not known [23]. Graphical models are a key element in this method. This result can be extended to binary variables, when interactions in underlying linear regressions are assumed to be absent. For a detailed explanation of this extension and the relation to linear regression, see the Appendix (Additional File 1).

As discussed before, in general intervention effects cannot be determined uniquely by observations alone. In such cases, one obtains a set of possible intervention effects which contains the true effect but might also contain wrong ones. This is a conceptual problem and occurs even if there are infinitely many observations. Using the CHC data, we analyzed the question: "Which ICF category has the most positive effect on the dependent variable General Health Perception (ghp) if it is improved by external intervention (e.g. therapy)?" To this end, we estimated the intervention effect of each ICF category on ghp for each of the 50 data sets obtained from multiple imputation and bootstrapping. Since the estimated intervention effect for each ICF category and in each data set need not be unique, we usually obtained more than 50 estimates of the intervention effect for each ICF category. We then aggregated the estimates for each ICF category using the mean over all estimates for one ICF category. We report on the ranking of the categories according to this measure of effect strength.

Estimation of the skeleton and the intervention effects was done using the R-package "pcalg".

## Results

### Visualization

**Figure 1:**
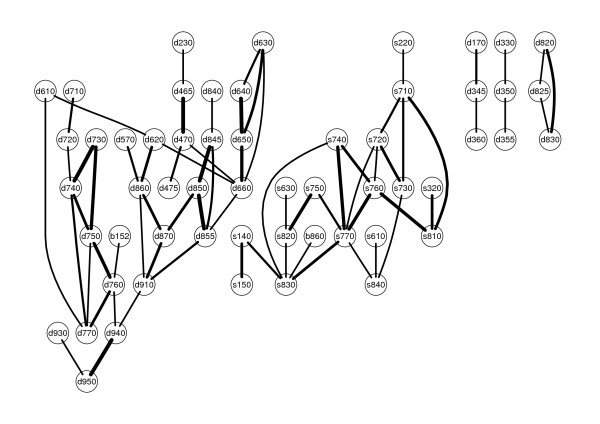
**Five largest connected components**. All five connected components containing at least three nodes in the skeleton computed on the SCI data.

The summary graph based on the variables of the SCI data set was computed. We show the five largest connected components in Figure 1.

For sake of brevity, we only present examples of strong associations in the largest connected component (on the left in Figure 1). There was a cluster of categories all concerned with relationships. *Informal social relationships (d750) *was associated with *Relating with strangers (d730)*, *Formal relationships (d740)*, *Intimate relationships (d770)*, and *Family relationships (d760)*. Formal relationships led to a small structure connecting *Complex interpersonal interactions (d720) *with *Basic interpersonal interactions (d710)*. *Family relationships (d760) *was also associated with a category from the component Body Functions, *Emotional functions (b152)*. To give another example, one path led from the category *Assisting others (d660) *to a triangle formed by *Preparing meals (d630)*, *Doing housework (d640) *and *Caring for household objects (d650)*. *Assisting others (d660) *also led to a triangle formed by *Non-remunerative employment (d855)*, *Remunerative employment (d850)*, and *Acquiring, keeping and terminating a job (d845)*, the latter also being associated with *Apprenticeship (d840)*.

### Dimension reduction

The resulting summary graph for the SCI data was split up into connected components. There were 5 connected components with at least 3 nodes involved, which are shown in Figure 1 (that the largest connected component was already discussed in the previous section). Thus, the original high dimensional structure was broken down into several independent, low dimensional structures.

Furthermore, the skeleton gave information on the dependence structure within each component.

### Comparison of subpopulations

**Figure 2:**
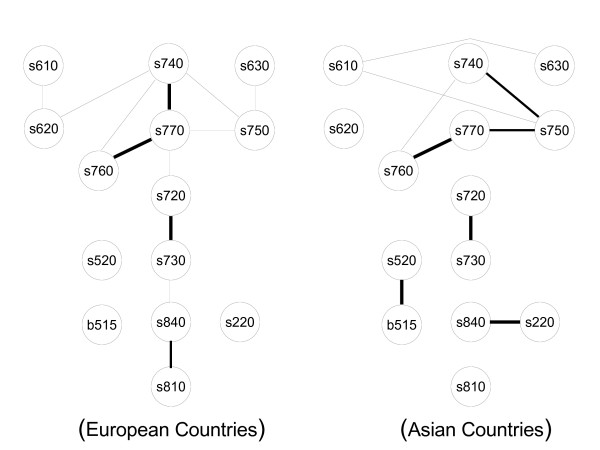
**Comparison of Asian and European countries**. Skeleton for European (left) and Asian (right) countries computed on the SCI data.

The dependence structure between ICF categories for Asian countries and European countries differed significantly. Figure 2 shows one distinct group of categories with the corresponding subgraphs in the European subsample (left) and the Asian subsample (right) of the SCI data. There are obvious differences. Using SHD as a measure of distance, the two subgraphs differed significantly, i.e., the variation between the two regions is larger than the variation introduced by both bootstrap and multiple imputation.

To give an example, *Structure of esophagus (s520) *was associated with *Digestive functions (b515) *in the Asian subsample but not in the European. Moreover, the association between *Additional musculoskeletal structures related to movement (s770) *and *Structure of pelvic region (s740) *was present in Europe but not in Asia.

### Estimating causal (intervention) effects

We predicted the top five categories that had the most beneficial effect on General Health Perception (ghp), when improved by external intervention, e.g., therapy. The category *Doing housework (d640) *had the strongest effect, followed by *Sensation of pain (b280)*, *Walking (d450)*, *Lifting and carrying objects (d430)*, and *Recreation and Leisure (d920)*.

## Discussion

We found that graphical models can be used in the study of ICF data in order to develop a more detailed understanding of human functioning.

Graphical models could be used for visualization of functioning in patients with spinal cord injury. The associations in Figure 1 have high face validity. Even though there were no a priori hypotheses imposed, the model revealed a clustering of variables concerning relationships and interpersonal interactions. The representation of the findings as graph is convenient, since it facilitates easy detection of groupings. Thus, this method might help to promote an intuitive understanding of human functioning.

The skeleton of a Directed Acyclic Graph is a somewhat refined version of the Conditional Independence Graph (CIG) used by [8]. The skeleton has the advantage that its edges indicate a stronger kind of dependence than the edges in a CIG. This is because the skeleton has an edge, if and only if the endpoints are dependent given any subset of the remaining nodes, whereas the CIG has an edge, if and only if the endpoints are dependent given all other nodes (but no subset is tested). Thus, edges in the CIG might vanish when conditioning on a subset of the remaining variables.

After estimating the skeleton, we found several connected components which can be used for dimension reduction. Dimension reduction allows focusing the analysis only on the part of the variables that are of interest. The connected components can be seen as distinct constructs and possibly be treated as one single variable in further analysis. This is useful, since many statistical methods are well suited for the case where many observations and few variables are available. Since dimension reduction transforms a large analysis task into (possibly one or many) smaller ones, these methods might only become feasible on the smaller groups. In particular, this is useful for dealing with ICF data. For example, when constructing a unidimensional scale for the difficulty of a task corresponding to nodes in the graph, fitting a Rasch model to a set of 200 variables seems daunting, whereas fitting it to several independent groups of five to twenty variables is much more feasible. Moreover, the structure of the connected components might yield additional information on the internal structure. Further analyses might benefit from this information. We found that the differences in the dependence structures between subpopulations were relevant and could be systematically analyzed using graphical models. Apparently, context seemed to matter, since the graphical models estimated for different geographical regions did vary significantly. Since the data used in this example is hardly representative, this cannot be discussed on the content level. Nevertheless, stratification and comparison of structures between strata can be very useful when developing theories about human functioning. Since graphical models represent complex structures in a well defined, mathematical way, they represent an ideal fundament for further methodological development on the systematic comparison of structures.

When estimating bounds on causal effects of ICF categories on general health perceptions, we found that the five ICF categories that showed the strongest effects were plausible. Four of the five categories found (*d640, d450, d430, d920*) are closely related to physical activity in daily life and it is plausible that general health perception is increased by increasing physical activity. Moreover, all categories we found are addressed in at least one of the currently most widely used health status measures [24]. The findings are in line with a previous study on the same data set (see [10]) using regression analysis (RA) instead of intervention analysis (IA). Four of the top five variables (*d640*, *b280*, *d450 *and *d920*) occur in both RA and IA. Moreover, *Emotional Functions (b152) *which appears important in RA is on rank 7 in IA and thus also found to be very influential. Hence, content-wise, there was a large overlap of the concepts found with RA and IA, which is quite encouraging. Drawing a conclusion, IA seems to extract different but in a broad sense related information from the data. From a conceptual point of view, we think that the approach of intervention calculus - by estimating causal effects - is preferable to the associational approach (i.e. regression analysis), since the effect of a therapy is a matter of causation and not of association. Thus, from a therapeutic perspective, a regression analysis might be misleading in a sense that it suggests variables for intervention which are not causally related to the outcome. In principle, estimating the intervention effect directly overcomes this problem and finds promising candidates for successful therapeutic intervention more efficiently. Understanding the impact of therapeutic interventions is valuable for both pragmatic therapeutic suggestion and for the understanding of human functioning in general. It is important to note that the results shown are meant to be proofs of principle. Medical conclusions or interpretations are not to be drawn, since the data sets used here were convenience samples not representative of the underlying population. Specifically, the choice of countries representing the stratification into Asia and Europe is by no means representative and was made to get subgroups with reasonable sample size. Additionally, there was no information on the potential informativeness of missing values which were imputed under the assumption of noninformative missingness. Variables were dichotomized since this was necessary for the proposed methods. Yet this encompasses a loss of information. A generalization of the methodology that includes categorical or even ordinal data would be desirable.

As with all statistical methods, errors might occur due to sampling, i.e., some edges might be missing and some edges might be superfluous. We addressed this problem by using the bootstrap. However, there is a need to develop more rigorous methods for assessing the reliability of the estimated graph.

As with many other statistical methods, graphical modeling is based on certain assumptions, whose validity is hard to check in practice. For all of our applications, we assumed the absence of hidden or selection variables. Furthermore, we assumed that it is possible to represent all true independent statements of a complex structure using a graphical model without making any error (this is often called "faithfulness" or "stability"). It is reasonable to assume that this is often the case (see [17]).

While graphical models contribute to dimension reduction, other methods might be superior in particular applications. In many situations involving graphical models, erroneous estimation of one edge is not crucial for the global result. However, if we use graphical models for dimension reduction, one misplaced edge might change the result completely. For example, if we imagine that in Figure 2 an edge was erroneously inserted between d660 and s630, the two large groups would be combined into only one larger group. In this case, we would wrongly conclude that there is a dependency between the two groups. Thus, when used for dimension reduction, graphical models are sensitive to errors.

In order to compare the structures of different regions, we fitted one graphical model per region and compared the graphical models using SHD. This comparison was based on heuristics. Further research has to be done in order to provide systematic and computationally feasible methods for detecting significant differences between graphs.

For estimating intervention effects, we additionally assumed that the true causal mechanism can be represented by a Directed Acyclic Graph, i.e. we assumed that there are no feedback loops. Furthermore, we introduced some restrictions on the dependence of the individual random variables since interactions among explanatory variables were assumed to be absent. Without making assumptions, no information on causal effects can be found. Under our assumptions, we can find sets of possible causal effects. Even when given an infinite amount of data and using our assumptions, it will in general not be possible to find a unique causal effect, but only sets of possible causal effects. The development of suitable methods for aggregation of ambiguous causal effects is desirable. Ideally, as a strong test of the underlying assumptions, one would compare the performance of our proposed method for causal inference with the outcomes of randomized experiments.

## Conclusions

Graphical models are a flexible modeling tool. They represent and quantify interaction between a possibly high number of active elements. Networks and graphs streamline multiple factors to information structures and are a starting point for gaining a more detailed understanding of a complex reality. Based on our results, graphs as studied in this paper (and networks in a more general understanding) may emerge as new paradigms to describe and understand complex structures representing the human experience and might be a milestone in developing a more detailed understanding of human functioning.

## Competing interests

The authors declare that they have no competing interests.

## Authors' contributions

MK, PB and MM developed the methodology. MK implemented the methods and analyzed the data sets. MK, BF, EG wrote the majority of the text. UM helped planning the outline of the paper and contributed some parts of the text. GS, PB and MM supervised the analysis of the data sets and helped in the detailed structuring and wording of the paper. All authors have read and approved the final manuscript.

## Pre-publication history

The pre-publication history for this paper can be accessed here:

http://www.biomedcentral.com/1471-2288/10/14/prepub

## Supplementary Material

Additional file 1 **Estimating causal (intervention) effects for binary data**. The method for estimating causal (intervention) effects using Gaussian data is adapted for binary data without interaction. Click here for file

## References

[B1] Bickenbach JE Chatterji S Badley EM Ustun TB Models of disablement, universalism and the international classification of impairments, disabilities and handicaps Social Science and Medicine 1999 48 9 1173 1187 10.1016/S0277-9536(98)00441-9 10220018

[B2] Zola IK Toward the necessary universalizing of a disability policy Milbank Quarterly 1989 67 401 428 10.2307/3350151 2534158

[B3] Stucki G Cieza A Melvin J The international classification of functioning, disability and health: A unifying model for the conceptual description of the rehabilitation strategy J Rehabilitation Medicine 2007 39 4 279 285 10.2340/16501977-0041 17468799

[B4] World Health Organization58th World Health Assembly, Resolution R114: disability, including prevention, management and rehabilitation. Adopted in may 2005. Geneva 2005

[B5] Frontera WR Fuhrer MJ Jette AM Chan L Cooper RA Duncan PW Kemp JD Ottenbacher KJ Peckham PH Roth EJ Tate DG Rehabilitation medicine summit - Building research capacity Am J Phys Medicine and Rehabilitation 2005 84 12 913 926 10.1097/01.phm.0000190316.61035.5e 16327406

[B6] Tsai CL Camargo CA Methodological considerations, such as directed acyclic graphs, for studying "acute on chronic" disease epidemiology: Chronic obstructive pulmonary disease example J Clinical Epidemiology 2009 62 982 990 10.1016/j.jclinepi.2008.10.005 19211222

[B7] Hernan MA Robins JM Instruments for causal inference - An epidemiologist's dream? Epidemiology 2006 17 4 360 372 10.1097/01.ede.0000222409.00878.37 16755261

[B8] Strobl R Stucki G Grill E Müller M Mansmann U Graphical models illustrated complex associations between variables describing human functioning J Clinical Epidemiology 2009 62 9 922 933 10.1016/j.jclinepi.2009.01.018 19540719

[B9] Biering-Sorensen F Scheuringer M Baumberger M Charlifue SW Post M Montero F Kostanjsek N Stucki G Developing core sets for persons with spinal cord injuries based on the International Classification of Functioning, Disability and Health as a way to specify functioning Spinal Cord 2006 44 9 541 546 10.1038/sj.sc.3101918 16955074

[B10] Cieza A Geyh S Chatterji S Kostanjsek N Ustun B Stucki G Identification of candidate categories of the International Classification of Functioning Disability and Health (ICF) for a Generic ICF Core Set based on regression modelling BMC Medical Research Methodology 2006 6 36 10.1186/1471-2288-6-36 16872536 PMC1569864

[B11] Ware JE Sherbourne CD The MOS 36-item short-form health survey (sf-36). 1. conceptual-framework and item selection Medical Care 1992 30 6 473 483 10.1097/00005650-199206000-00002 1593914

[B12] The R Project for Statistical Computing http://www.r-project.org

[B13] van Buuren S Multiple imputation of discrete and continuous data by fully conditional specification Statistical Methods In Medical Research 2007 16 3 219 242 10.1177/0962280206074463 17621469

[B14] Ibrahim JG Chen MH Lipsitz SR Herring AH Missing-data methods for generalized linear models: A comparative review J Am Statistical Association 2005 100 469 332 346 10.1198/016214504000001844

[B15] Hastie T Tibshirani R Friedman J The elements of statistical learning 2009 2 Springer Series in Statistics

[B16] Lauritzen SL Graphical models 1996 Oxford University Press

[B17] Pearl J Causality 2000 Cambridge University Press

[B18] Kalisch M Bühlmann P Estimating high-dimensional directed acyclic graphs with the PC-algorithm J Machine Learning Research 2007 8 613 636

[B19] Spirtes P Glymour C Scheines R Causation, Prediction, and Search 2000 2 The MIT Press

[B20] Tsamardinos I Brown LE Aliferis CF The max-min hill-climbing Bayesian network structure learning algorithm Machine Learning 2006 65 1 31 78 10.1007/s10994-006-6889-7

[B21] Cieza A Geyh S Chatterji S Kostanjsek N Ustun B Stucki G Identification of candidate categories of the International Classification of Functioning Disability and Health (ICF) for a Generic ICF Core Set based on regression modelling BMC Medical Research Methodology 2006 6 36 10.1186/1471-2288-6-36 16872536 PMC1569864

[B22] J Ramsey JZ Spirtes P Adjacency-Faithfulness and Conservative Causal Inference Uncertainty in Artificial Intelligence 2006

[B23] Maathuis MH Kalisch M Bühlmann P Estimating high-dimensional intervention effects from observational data Annals of Statistics 2009 37 3133 3164 10.1214/09-AOS685

[B24] Cieza A Stucki G Content comparison of health-related quality of life (HRQOL) instruments based on the international classification of functioning, disability and health (ICF) Quality Life Research 2005 14 5 1225 1237 10.1007/s11136-004-4773-0 16047499

