# Supplementary material for: Understanding human functioning using graphical models
Source: BMC Med Res Methodol. 2010 Feb 11;10:14. doi: 10.1186/1471-2288-10-14 (PMC2831907; doi:10.1186/1471-2288-10-14)
Supplement: Additional file 1 — Estimating causal (intervention) effects for binary data. The method for estimating causal (intervention) effects using Gaussian data is adapted for binary data without interaction. [file 1471-2288-10-14-S1.PDF]

Estimating causal (intervention) effects for binary data

In [1], the estimation of causal (intervention) effects for Gaussian data was discussed and fast algorithms were proposed. In this text, we adapt these results for binary data. For the notation concerning causal effects, refer to [2].

Let $X_1, \dots, X_p$ be p binary explanatory variables and let Y be a binary dependent variable. Without loss of generality, all levels are assumed to be in $\{0, 1\}$. We define the causal effect of X_i on Y by

$$E(Y|do(X_i = 1)) - E(Y|do(X_i = 0)).$$

Let G be the directed acyclic graph on $\{X_1, \dots, X_p, Y\}$ describing the true underlying causal structure. (In practice, this graph is estimated.) Let Pa_i denote the parents of X_i in G.
For $Pa_i = \emptyset$, back-door adjustment (see Theorem 3.3.2 in [2]) implies that the causal effect of X_i on Y is

$$E(Y|do(X_i = 1)) - E(Y|do(X_i = 0)) = E(Y|X_i = 1) - E(Y|X_i = 0), \quad (1)$$

as illustrated in the following coordinate system.

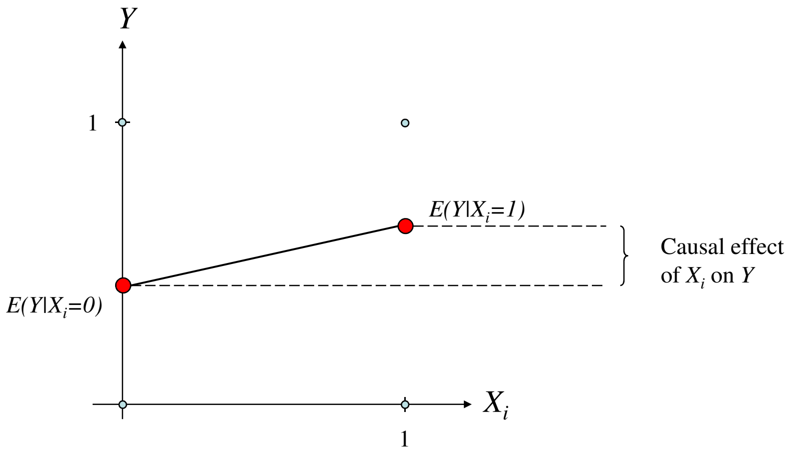

Figure 1: Illustration of the causal effect in the binary case when $Pa_i = \emptyset$.

Since the horizontal distance in Figure (1) is equal to 1, the causal effect is exactly the same as the slope of the straight line through the points $(0, E(Y|do(X_i = 0)))$ and $(1, E(Y|do(X_i = 1)))$. For any $S \subseteq \{X_1, \dots, X_p\} \setminus \{X_i\}$, let $\beta_{i|S}$ denote the

regression coefficient of X_i in the regression of Y on X_i and S. Then, the causal effect defined in equation (1) is given by $\beta_{i|\emptyset}$, i.e., the regression coefficient of X_i in the regression of Y on X_i.
For $Pa_i = \{X_j\}$, the causal effect of X_i on Y is

$$\begin{aligned}
& E(Y|do(X_i = 1)) - E(Y|do(X_i = 0)) \\
= & \sum_{x_j} E(Y|X_i = 1, X_j = x_j)P(X_j = x_j) - \sum_{x_j} E(Y|X_i = 0, X_j = x_j)P(X_j = x_j) \\
= & [E(Y|X_i = 1, X_j = 1)P(X_j = 1) + E(Y|X_i = 1, X_j = 0)P(X_j = 0)] \\
& -[E(Y|X_i = 0, X_j = 1)P(X_j = 1) + E(Y|X_i = 0, X_j = 0)P(X_j = 0)] \\
= & [E(Y|X_i = 1, X_j = 1) - E(Y|X_i = 0, X_j = 1)]P(X_j = 1) \qquad (2) \\
& +[E(Y|X_i = 1, X_j = 0) - E(Y|X_i = 0, X_j = 0)]P(X_j = 0).
\end{aligned}$$

If there is no interaction between X_i and X_j, we have

$$\begin{aligned}
& E(Y|X_i = 1, X_j = 1) - E(Y|X_i = 0, X_j = 1) \\
& \quad = E(Y|X_i = 1, X_j = 0) - E(Y|X_i = 0, X_j = 0) = \beta_{i|X_j}.
\end{aligned}$$

Plugging this into equation (2) yields that the causal effect of X_i on Y is

$$\beta_{i|X_j} P(X_j = 1) + \beta_{i|X_j} P(X_j = 0) = \beta_{i|X_j}.$$

If Pa_i contains several variables, one can show analogously that the causal effect of X_i on Y is given by $\beta_{i|Pa_i}$. If there is interaction, this should be taken into account in the regression. However, in the case with interaction, many more parameters have to be estimated and this might lead to accuracy problems. Thus, one has to weigh the advantage of using interactions against the disadvantage of badly estimated parameters.

References

[1] Maathuis MH, Kalisch M, Bühlmann P: **Estimating high-dimensional intervention effects from observational data**. *Annals of Statistics* 2009, **37**:3133–3164.

[2] Pearl J: *Causality.* Cambridge University Press 2000.
